# Trends in Violent Penetrating Injuries During the First Year of the COVID-19 Pandemic

**DOI:** 10.1001/jamanetworkopen.2021.45708

**Published:** 2022-02-08

**Authors:** Elizabeth C. Pino, Erika Gebo, Elizabeth Dugan, Jonathan Jay

**Affiliations:** 1 Boston Violence Intervention Advocacy Program, Department of Emergency Medicine, Boston Medical Center, Boston, Massachusetts; 2 Department of Sociology & Criminal Justice, Suffolk University, Boston, Massachusetts; 3 Boston Violence Intervention Advocacy Program, Department of Emergency Medicine, Boston Medical Center, Boston, Massachusetts; 4 Department of Community Health Sciences, Boston University School of Public Health, Boston, Massachusetts

## Abstract

**Importance:**

Public health measures instituted to reduce the spread of COVID-19 led to severe disruptions to the structure of daily life, and the resultant social and financial impact may have contributed to an increase in violence.

**Objective:**

To examine the trends in violent penetrating injuries during the first COVID-19 pandemic year compared with previous years.

**Design, Setting, and Participants:**

This retrospective cross-sectional study was performed to compare the prevalence of violent penetrating injuries during the first COVID-19 pandemic year, March 2020 to February 2021, with the previous 5 years, March 2015 to February 2020. This study was performed among all patients with a violent penetrating injury presenting at Boston Medical Center, an urban, level I trauma center that is the largest safety-net hospital and busiest trauma center in New England. Data were analyzed from January 4 to November 29, 2021.

**Main Outcomes and Measures:**

The primary outcomes were the incidence and timing of emergency department presentation for violent penetrating injuries during the first year of the COVID-19 pandemic compared with the previous 5 years. Patient demographics and injury characteristics were also assessed.

**Results:**

A total of 2383 patients (median [IQR] age, 29.5 [23.4-39.3] years; 2032 [85.4%] men and 351 [14.6%] women) presenting for a violent penetrating injury were evaluated, including 1567 Black patients (65.7%), 448 Hispanic patients (18.8%), and 210 White patients (8.8%). There was an increase in injuries during the first pandemic year compared with the previous 5 years, with an increase in shootings (mean [SD], 0.61 [0.89] injuries per day vs 0.46 [0.76] injuries per day; *P* = .002) but not stabbings (mean [SD], 0.60 [0.79] injuries per day vs 0.60 [0.82] injuries per day; *P* = .78). This surge in firearm violence began while Massachusetts was still under a stay-at-home advisory and before large-scale racial justice protests began. Patients presenting with violent penetrating injuries in the pandemic surge months (April-October 2020) compared with the same period in previous years were disproportionately male (153 patients [93.3%] vs 510 patients [87.6%]; *P* = .04), unemployed (70 patients [57.4%] vs 221 patients [46.6%]; *P* = .03), and Hispanic (40 patients [26.0%] vs 99 patients [17.9%]; *P* = .009), with a concurrent decrease in White patients (0 patients vs 26 patients [4.7%]), and were more likely to have no previous history of violent penetrating injury (146 patients [89.0%] vs 471 patients [80.9%]; *P* = .02).

**Conclusions and Relevance:**

These findings suggest that unprecedented measures implemented to mitigate the spread of COVID-19 were associated with an increase in gun violence. As the pandemic abates, efforts at community violence prevention and intervention must be redoubled to defend communities against the epidemic of violence.

## Key Points

**Question:**

Was the COVID-19 pandemic associated with differences in levels, timing, and types of violent penetrating injuries presenting to a level I urban trauma center?

**Findings:**

In this cross-sectional study that included 2383 patients with violent injuries, there was a significant increase in patients who presented with firearm injuries but not stabbing injuries during the first pandemic year compared with the previous 5 years. This surge in firearm injuries began while Massachusetts was still under a stay-at-home advisory and before large-scale racial justice protests.

**Meaning:**

These findings suggest that policies and procedures aimed at mitigating firearm violence as part of all-hazard preparedness are warranted.

## Teaser

This cross-sectional study examines trends in violent penetrating injuries treated at an urban level I trauma center during the first year of the COVID-19 pandemic vs the previous 5 years.

## Introduction

In March of 2020, the full severity and transmissibility of SARS-CoV-2 became apparent in the United States.^1,2^ The virus had already been circulating widely in Wuhan, China, and Bergamo, Italy, leading to extreme levels of morbidity and mortality due to respiratory failure and ultimately a collapse of overburdened health care systems.^3,4^ Unprecedented public health measures were taken in most US states to prevent a similar scenario.^5^ Schools and businesses were forced to substitute in-person activities with remote learning and virtual meetings, and some nonessential industries paused operations indefinitely.^6^ Massachusetts issued a stay-at-home advisory from March 24, 2020, through May 18, 2020, that shut down all nonessential businesses and urged residents not to go out except to do essential activities or take a walk.^7^

These measures curbed the spread of COVID-19,^8^ but the resultant societal impacts may have contributed to an unexpected increase in violence.^9,10^ Trauma admissions for violent intentional injuries increased to the highest levels in years,^9,10,11,12,13^ and police department data revealed substantial increases in serious violence since the start of the pandemic.^14^ However, numerous uncertainties remain as to the nature of this violence spike, including the importance of firearms,^11,14,15^ the role of stay-at-home orders,^16^ and social unrest in the wake of George Floyd’s murder by Minneapolis, Minnesota, police.^14,17,18^ The burden of additional violence disproportionately affected communities of color, such as Black and Hispanic communities, such as that reported in Philadelphia, Pennsylvania,^11^ but it is unclear how these disparities were associated with racial and economic marginalization, changes to daily activities, and other factors. Moreover, much of the existing literature is based only on patterns during the initial months of the pandemic,^11,12,15,16^ and disproportionately documents patterns from only 1 US city (ie, Philadelphia).^11,12,16^

Understanding the nature of violence during COVID-19 is necessary for allocating resources to prevent violence and respond to its aftereffects. Furthermore, if violent injury trends have changed, then traditional prevention and intervention practices may need to be reexamined or redirected to ensure that those most in need of services are adequately being reached. In this study, we examined changes in presentation for violent penetrating injuries at the emergency department (ED) of an urban, level I trauma center and safety-net hospital in Boston, Massachusetts, throughout the first year of the COVID-19 pandemic. In particular, our objectives were to assess (1) the magnitude of the increase in firearm injuries, compared with previous years and compared with changes in other violent penetrating injuries; (2) the timing of the increase in firearm violence and its association with daily activity patterns, given the stay-at-home orders and racial protests; and (3) the demographic and socioeconomic characteristics of patients presenting with firearm injury during the pandemic.

## Methods

The Boston University, Boston Medical Center (BMC) institutional review board approved this study with a waiver of Health Insurance Portability and Accountability Act of 1996 authorization for research and deemed this study exempt from federal regulations for the protection of human research participants. Informed consent was waived because it was deemed impracticable for retrospectively collected clinical data, given the large sample size and difficulty of relocating patients. Results were reported according to the Strengthening the Reporting of Observational Studies in Epidemiology (STROBE) reporting guideline.

### Study Design

This retrospective cross-sectional study was performed using electronic health record data^19^ from a cohort of patients presenting to the BMC ED for a violent penetrating injury between 2015 and 2021. BMC, the region’s largest safety-net hospital, is a level I trauma center that treats approximately 50% to 70% of patients with gunshot or stabbing injuries in the city of Boston (eTable 1 in the Supplement).^20^ This data set captures all penetrating injuries severe enough to be treated in the ED, in contrast to police department data, which only capture critical injuries requiring a response from law enforcement. All patients with penetrating injuries due to community or interpersonal violence from March 2015 to February 2021 were included in this analysis. Data were obtained from the BMC Violence Intervention Advocacy Program (VIAP) clinical database. Visits for violent injuries were identified by self-report; VIAP trauma response personnel who respond 24 hours, 7 days a week to BMC for immediate crisis intervention; attending physician assessment, evaluation, and diagnosis; or Boston Police Department notification. Disagreements regarding whether an injury was self-inflicted, accidental, or due to community or interpersonal violence were resolved based on assessment by a clinical team of social workers, VIAP violence intervention advocates, and physicians. Injuries deemed to be self-inflicted were excluded. This data set was merged with state public health data of daily COVID-19 hospitalizations.

### Measures

Data on the 7-day mean of COVID-19 hospitalizations in Massachusetts were acquired from the Massachusetts Department of Public Health COVID-19 Data Dashboard.^21^ To assess the association of societal events during the COVID-19 pandemic, we used the dates that the state’s stay-at-home advisory went into effect (March 24, 2020),^7^ the date it was lifted (May 18, 2020), and the date that large-scale protests began (May 28, 2020)^22^ in response to police and vigilante violence against Black individuals in the US. The period of the stay-at-home advisory was used as a proxy measure for a period of increased social distancing and staying at home, as well as financial strain among communities most at risk for violence,^23^ while the period after George Floyd’s death was used as proxy for a time of decreased social distancing and increased social unrest. Assessed variables are detailed in eAppendix 1 in the Supplement.^24^ Race and ethnicity were self-reported. These variables were included in analysis because of the well-documented racial and ethnic disparities in violent injuries.^25^

### Statistical Analysis

First, we compared yearly frequencies of violent injuries by mechanism of injury. To correspond with the timing of the first year of pandemic-related societal disruption (March 2020 through February 2021), all years reported begin in March of the designated year, and end in February of the following year. Injuries per day of the first pandemic year were compared with the previous 5 years using the Wilcoxon rank sum test. Based on the results of this preliminary analysis, we focused the remaining analyses on firearm injuries. Consistent with best practices for interrupted time series analysis,^26,27^ interventional autoregressive integrated moving average (ARIMA) models were used to quantify the association of the pandemic and social justice movements with observed monthly firearm injuries, detailed in eAppendix 2 in the Supplement.^28,29,30^

To assess daily temporal changes in firearm injuries, our third analysis used kernel-weighted local polynomial regression^31,32^ to observe local mean smoothed graphs of the 7-day mean of penetrating injuries per day. We used the findings from this step to identify contiguous surge months in 2020, during which the 7-day moving mean of daily firearm injuries exceeded rates from the previous 5 years. The remaining analyses assessed trends during the surge months of 2020 compared with the same periods in the previous 5 years, detailed in eAppendix 3 in the Supplement.^33^

All analyses were conducted in Stata statistical software version 16 (StataCorp). All statistical tests used 2-sided *P* < .05 as the threshold for significance. Data were analyzed from January 4 to November 29, 2021.

## Results

**Figure 1.**
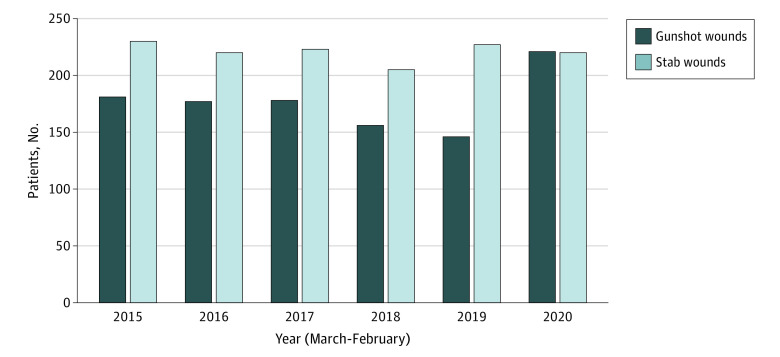
Annual Frequencies of Individuals With Penetrating Injuries Treated at Boston Medical Center, 2015-2021

A total of 2383 patients (median [IQR] age, 29.5 [23.4-39.3] years; 2032 [85.4%] men and 351 [14.6%] women) presenting for a violent penetrating injury were evaluated from 2015 to 2021, including 1567 Black patients (65.7%), 448 Hispanic patients (18.8%), and 210 White patients (8.8%). During the first year of the pandemic, we found an increase in penetrating injuries compared with the previous 5 years (Figure 1; eTable 2 in the Supplement). There were 441 patients with penetrating injuries in the first year of the COVID-19 pandemic (ie, March 2020-February 2021), compared with a mean (SD) of 388 (19) patients per year in the previous 5 years. This increase was exclusively due to an increase in gunshot wounds during the COVID-19 pandemic compared with previous years (mean [SD], 0.61 [0.89] injuries per day vs 0.46 [0.76] injuries per day; *P* = .002), whereas there was no change in stab wounds (mean [SD], 0.60 [0.79] injuries per day vs 0.60 [0.82] injuries per day; *P* = .78). The 221 firearm injuries during the pandemic year constituted a 32% increase from the previous 5 years, and a 51% increase from the year beginning in March of 2019.

**Figure 2.**
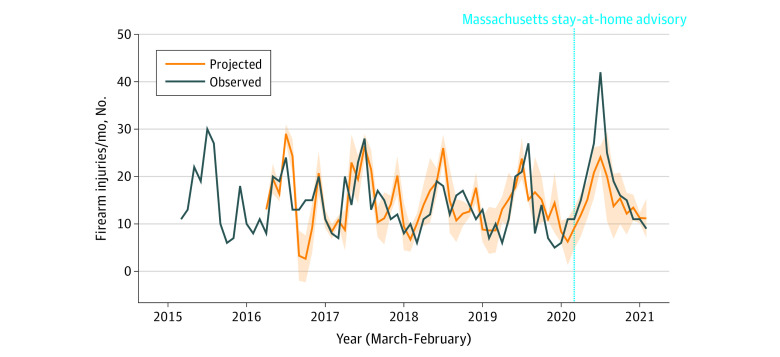
Observed and Projected Firearm Injuries per Month Treated at Boston Medical Center, 2015-2020 Shading indicates 95% CI of projected injuries.

The ARIMA model that best fit the monthly data of firearm injuries is presented in Figure 2, with an interrupted time series with March 2020 designating the start of the Massachusetts stay-at-home advisory. We observed a significant increase in firearm injuries from May to July 2020 compared with the projected model (eg, July: 42 actual injuries vs 24.1 [95% CI, 21.7-26.5] projected injuries). The time series trend increased by 13.1 (95% CI, 6.94-19.28; *P* < .001) (ω0) firearm injuries per month after the intervention, and then decayed by (ω × δ^k^), in which *k* represents each consecutive time point (δ = 0.87 [95% CI, 0.78-0.97]; *P* < .001).

**Figure 3.**
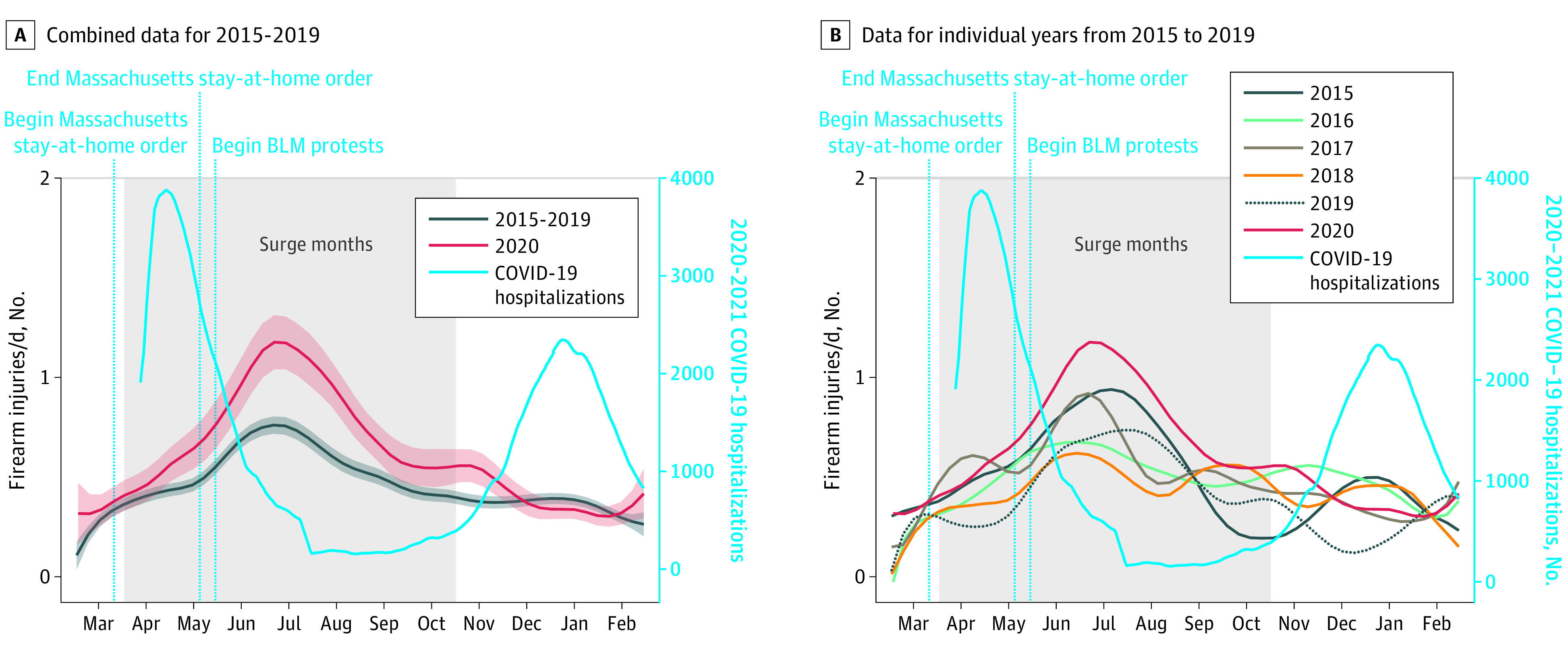
Smoothed Trends in Daily Firearm Injuries Treated at Boston Medical Center, 2015-2021 A, Construction of 95% CIs for local mean smoothed graphs of the 7-day mean of penetrating injuries per day was performed by computing SEs obtained by taking a square root of the estimate of the conditional variance of the local polynomial estimator at each day of the year. BLM indicates Black Lives Matter; shading, 95% CI.

To better understand trends in association with societal disruptions in the pandemic year, we modeled a smoothed estimate of daily firearm injuries (Figure 3; eFigure 1 in the Supplement). We observed an increase in firearm injuries while Massachusetts was still under a stay-at-home advisory, between March 24 and May 18, 2020, compared with the same period in previous years. This increase was visible well before the onset of the Black Lives Matter (BLM) protests in response to the murder of George Floyd and other incidents of racist violence perpetrated by police or vigilantes, which began on May 28, 2020, in Boston. Although the increase coincided with the peak of hospitalizations due to COVID-19, hospitalizations then sharply declined, but ED visits for firearm injuries did not. Pandemic-year gun violence did not decrease back to the levels of previous years until November 2020. At that time, COVID-19 hospitalizations increased again, and firearm injuries remained at typical levels during the second wave of COVID-19.

**Figure 4.**
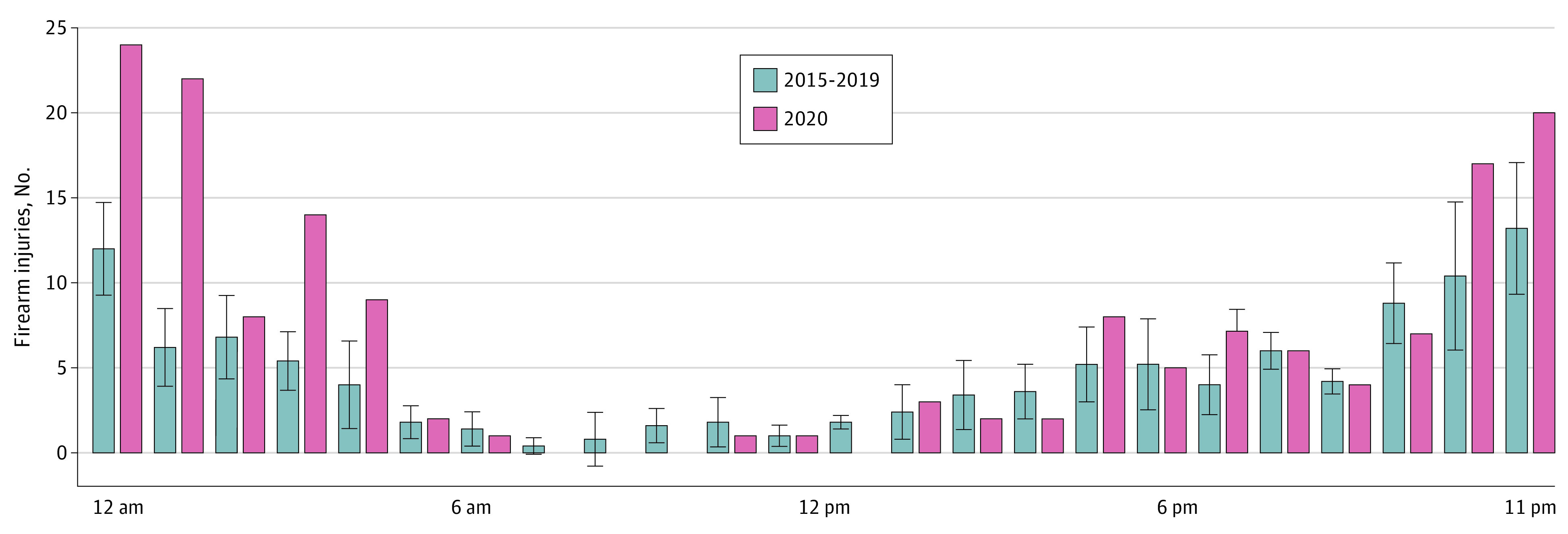
Trends in Triage Time for Firearm Injuries Treated at Boston Medical Center Between April and October, 2015-2020 Data for 2015 to 2020 are shown as means and 95% CIs (whiskers).

Despite dramatic changes to school and work schedules during the COVID-19 pandemic, firearm injuries during the surge months occurred primarily during the same late-night hours in 2020 as in previous years, albeit at higher levels (Figure 4). However, firearm injuries in 2020 occurred in higher numbers on Tuesdays through Thursdays and on weekends (eFigure 2 in the Supplement).

Patient demographics and injury characteristics of patients presenting with gunshot wounds changed during the surge months of April to October 2020 compared with the same period in previous years (Table). Patients presenting with gunshot wounds in 2020, compared with the previous 5 years, were more likely to be male (153 patients [93.3%] vs 510 patients [87.6%]; *P* = .041) and Hispanic (40 patients [26.0%] vs 99 patients [17.9%]; *P* = .009). We found a decrease in firearm injuries among White patients during the same period (0 patients [0%] vs 26 patients [4.7%]). However, despite pandemic-related disruptions, Black individuals continued to carry most of the burden of firearm injuries. During the 2020 surge months, patients presenting with gunshot wounds were also more likely to be unemployed compared with previous years (70 patients [57.4%] vs 221 patients [46.6%]; *P* = .03). Patients presenting with gunshot wounds in 2020 had a significantly shorter length of stay (median [IQR], 1.7 [0.8-4.8] vs 2.7 [0.8-7.8] days; *P* = .04) and were less likely to have been previously treated for a violent penetrating injury at our trauma center compared with patients from previous years (18 patients [11.0%] vs 111 patients [19.1%]; *P* = .02). All variables found to be significantly different in 2020 compared with the previous 5 years were equivalent across 2015 to 2019 (eTable 3 in the Supplement). No significant differences were observed by age, insurance payer, housing status, hospital disposition, or discharge placement.

**Table.** Patient and Injury Characteristics of Patients With Firearm Injury Treated at Boston Medical Center Between April and October, 2015-2020

Characteristic	No. (%)						*P* value [excluding unknown]^a^
	2015	2016	2017	2018	2019	2020	
Patients, No.	127	112	130	104	108	164	.003
Age, y							
Median (IQR)	25.0 (21.9-31.7)	24.6 (21.6-32.6)	26.3 (21.6-34.1)	27.9 (22.7-33.9)	28.6 (22.7-34.6)	27.2 (22.0-33.8)	.88
≤27	76 (59.8)	71 (63.4)	69 (53.1)	47 (45.2)	45 (41.7)	79 (48.2)	.79
≥28	51 (40.2)	41 (36.6)	61 (46.9)	57 (54.8)	63 (58.3)	85 (51.8)	
Gender							
Male	112 (88.2)	93 (83.0)	111 (85.4)	97 (93.3)	96 (88.9)	153 (93.3)	.04
Female	15 (11.8)	19 (17.0)	19 (14.6)	7 (6.7)	12 (11.1)	11 (6.7)	
Race and ethnicity							
Black	103 (82.4)	73 (68.9)	98 (77.8)	69 (70.4)	73 (75.3)	110 (71.4)	.02 [.009]
Hispanic	18 (14.4)	24 (22.6)	21 (16.7)	23 (23.5)	13 (13.4)	40 (26.0)	
White	4 (3.2)	6 (5.7)	6 (4.8)	5 (5.1)	5 (5.2)	0	
Other^b^	0	3 (2.8)	1 (0.8)	1 (1.0)	6 (6.2)	4 (2.6)	
Unknown	2	6	4	6	11	10	
Insurance payer							
Medicaid or Medicare	68 (70.1)	67 (76.1)	81 (75.7)	40 (54.8)	44 (57.9)	69 (66.4)	.01 [.73]
Private	11 (11.3)	14 (15.9)	12 (11.2)	15 (22.6)	22 (29.0)	16 (15.4)	
No health insurance	18 (18.6)	7 (8.0)	14 (13.1)	18 (24.7)	10 (13.2)	19 (18.3)	
Unknown	30	24	23	31	32	60	
Housing status							
Permanent home	94 (89.5)	94 (93.1)	106 (90.6)	69 (79.3)	68 (87.2)	110 (90.9)	.008 [.42]
Homeless or group home^c^	11 (10.5)	7 (6.9)	11 (9.4)	18 (20.7)	10 (12.8)	11 (9.1)	
Unknown	22	11	13	17	30	43	
Employment							
Employed	57 (56.4)	46 (47.9)	58 (50.0)	41 (49.4)	51 (65.4)	52 (42.6)	.02 [.03]
Unemployed	44 (43.6)	50 (52.1)	58 (50.0)	42 (50.6)	27 (34.6)	70 (57.4)	
Unknown	26	16	14	22	30	42	
Reinjury	29 (22.8)	18 (16.1)	24 (18.5)	22 (21.2)	18 (16.7)	18 (11.0)	.02
Hospital disposition							
Admitted	92 (73.0)	91 (82.0)	99 (76.2)	72 (69.2)	77 (72.6)	108 (66.3)	.06
Discharged	28 (22.2)	16 (14.4)	25 (19.2)	17 (16.4)	18 (17.0)	34 (20.9)	
Discharged against medical advice	1 (0.8)	3 (2.7)	2 (1.5)	4 (3.9)	2 (1.9)	4 (2.5)	
Deceased	5 (4.0)	1 (0.9)	4 (3.1)	11 (10.6)	9 (8.5)	17 (10.4)	
Missing	1	1	0	0	2	1	
Length of stay for admitted patients, median (IQR), d	1.9 (0.75-7.65	1.7 (0.5-6.5)	3.4 (0.9-8.5)	4.0 (1.5-10.1)	3.6 (1.2-8.4)	1.7 (0.8-4.8)	.04
Discharge placement							
Home	73 (81.1)	76 (83.5)	82 (82.8)	66 (91.7)	63 (81.8)	88 (83.0)	.48
Rehabilitation or long-term care facility or further hospitalization	14 (15.6)	7 (7.7)	11 (11.1)	4 (5.6)	7 (9.1)	7 (6.6)	
Left against medical advice	2 (2.2)	5 (5.5)	2 (2.0)	1 (1.4)	3 (3.9)	6 (5.7)	
Police custody	0	1 (1.1)	1 (1.0)	0	1 (1.3)	1 (0.9)	
Deceased	1 (1.1)	2 (2.2)	3 (3.0)	1 (1.4)	3 (3.9)	4 (3.8)	
Missing	2	0	0	0	0	2	

^a^ *P* value excluding unknown category is shown in brackets. Categorical variables were compared using χ^2^ tests, except for hospital disposition and discharge placement, which were compared using Fisher exact test. Continuous variables were compared using the Wilcoxon rank sum test. All *P* values represent comparison of data from 2020 vs the previous 5 years combined.

^b^ Other race includes Asian, American Indian or Alaska Native, and Native Hawaiian or other Pacific Islander.

^c^ Homeless housing status includes patients living on the street, in a shelter, at friends’ houses, or unknown homeless location.

## Discussion

This cross-sectional study found that serious violence increased significantly during the COVID-19 pandemic in Boston, as measured by BMC trauma center visits. The increase in violence began at the peak of the first wave of COVID-19 hospitalizations and before the state’s stay-at-home advisory was lifted but well before large-scale protests for racial justice in the aftermath of the George Floyd killing. We also found that violence during the pandemic was more likely to involve firearms than prepandemic violence. Injury incidence by time of day remained similar to prepandemic patterns but increased on weekdays. Our data show that pandemic firearm injuries were disproportionately among Black men, and that Hispanic men compromised a larger share of patients presenting with gunshot wounds than they had prepandemic. Pandemic patients presenting with violent penetrative injuries were less likely to have presented before with penetrating injury than in prepandemic years and more likely to be unemployed at the time of injury.

Our finding that firearm injuries increased over the first pandemic year, while other penetrating injuries did not, is consistent with other studies examining the initial months of the pandemic.^9,11,12^ Neither our study nor reports from other trauma centers noted a concomitant increase in stabbing injuries during the pandemic year.^9,11,12^ This was a surprising result, as patients presenting with stab wounds often present with multiple other stressors. These patients are more likely to be experiencing homelessness or to have a substance use disorder,^34^ and these groups were adversely impacted during the pandemic. Additionally, social distancing restrictions implemented at homeless shelters reduced capacity, forcing individuals experiencing homelessness out onto the streets, and many people struggling with drug addiction were unable to receive treatment early in the pandemic.^35,36^ Individuals with more minor piercing injuries may have been less likely to present at the trauma center during the pandemic,^37^ yet it is unclear why the consequent increase in stress among this population was not associated with an increase in stabbing violence similar to the surge of firearm violence.

We found that firearm injuries began to surge before George Floyd’s killing spawned a sweeping BLM social justice movement opposing systemic anti-Black racism and structural violence. This finding generally runs counter to the proposal that changes in police activity due to protest movements explain the pandemic-year spike in firearm violence, at least in Boston.^38,39,40,41^ Our findings provide mixed support for the argument that changes in daily activities account for the change in firearm injuries. Although shootings increased during weekdays compared with previous years, they continued to occur primarily during overnight hours. It may be that people who, prepandemic, may have otherwise gone to work, completed schoolwork, or engaged with community resources that were altered or suspended during the first year of COVID-19, changed their daily patterns and were more exposed to potentially violent situations. We could not assess that proposition with our data. The consistency in late-night hours, but with high levels on most days of the week, points to the need to ensure not only that violence prevention services are amply available, but that immediate support and interventions are accessible when violence occurs, regardless of day or time.

During the pandemic, racial disparities affecting Black and Hispanic boys and men remained the same or worsened.^23,42^ Similarly, in our data, patterns of experiencing violence among Black and Hispanic boys and men during the pandemic year surge are consistent with an interpretation that pandemic violence exposure was primarily structured by racial and economic marginalization. In our population, we found that the increase in gun violence injuries occurred disproportionately among unemployed patients, men, and Hispanic patients, with a concurrent decrease among White patients; however, Black patients still constituted more than 70% of firearm injuries. A study of patients in trauma departments in Philadelphia similarly found the increase in injuries to be concentrated among younger males of color, including Black and Hispanic boys and men.^11^

Strain theory can support these findings. The COVID-19 pandemic, violent injuries, and homicides have disproportionately impacted neighborhoods of Black, Hispanic, and other racially marginalized individuals with lower socioeconomic position, reflecting a legacy of systemic racism and health inequities.^42^ The social and economic fallout from the pandemic was felt most acutely in these communities.^23,43^ In Boston, the unemployment rate increased sharply during lockdown, from 2.6% up to 16.1% by June but declined to 7.0% by February of 2021.^44^ Black and Hispanic households were more likely to experience job or wage loss owing to the COVID-19 pandemic and were less likely to have emergency financial reserves to cover basic monthly expenses.^23^ These same disparities could account for the increase in firearm violence during the pandemic year. Moreover, we found a lower rate of injury recidivism among patients presenting with firearm injuries in 2020, suggesting the possibility that increasing violence incidence ensnared individuals who shared common risk factors with individuals who had experienced violence before the pandemic but who had not been impacted as directly by cycles of community violence.

The curtailing of criminal justice functions, such as the closing of courts, more limited policing functions, and decarceration, have been cited as possible reasons for violence increases.^38,39,40^ However, this does not explain why there was an increase in shootings without concomitant increases in other crimes in Boston.^45^ One factor that may have contributed to the surge in violence is gun availability.^12,13,46,47^ An analysis of guns recovered by the Boston Police Department reported that most firearms were from out-of-state sources, especially New Hampshire, Maine, and Southern coastal states.^48^ These states, along with Massachusetts, experienced increases in firearm sales during the first year of the pandemic.^49,50^ It may be that individuals and groups, such as gangs, involved in criminal enterprise had easier access to sources of guns during the pandemic. Other possible influences include an increase in gang-related conflict^11,25,51^ and increased consumption of alcohol and other substances.^13,52^ However, our data on patients with traumatic injuries do not allow us to assess the potential role of any of these factors. Collecting and mapping these data onto injury trends is a task of future research that would benefit from a multiagency approach, like the Cardiff Model, which uses police data and collaborates with community partners in the violence prevention process.^53^

### Limitations

This study has several limitations. First, our population included patients from a single medical center, which limits the generalizability of our findings to other patient populations in other US cities and limits our knowledge of violent reinjury. This medical center treats approximately 50% to 70% of patients (approximately 400 patients per year) in the greater Boston area presenting for penetrating wounds. Second, as a retrospective study, we had to use proxy measures for some variables. The frequency of COVID-19 hospitalizations, indicative of the severity of the COVID-19 pandemic, and the timing of the BLM protests were used as general proxies for financial strain, social stress, and physical distancing among individuals during the study period. However, previous research suggests that state advisory orders were only associated with small increases in staying home in low-income neighborhoods, owing, in part, to the prevalence of occupations requiring residents to work outside the home.^54^ Third, while we were able to capture information regarding patient reinjury from hospital medical records, injuries not treated at BMC were not captured in this analysis. Fourth, the limitations of using of electronic health records to categorize demographics and injury specifics include the potential for missing, miscategorized, or incomplete data and inconsistencies in coding health record information.

## Conclusions

This cross-sectional study found that unprecedented social restriction measures implemented to mitigate the spread of COVID-19 were associated with an increase in firearm injuries in an urban setting. In Boston, the surge in firearm injuries began while Massachusetts was still under a stay-at-home advisory but before the start of the BLM protest movements. Despite dramatic changes to school and work schedules for most residents, firearm injuries during the surge months of April to October occurred during the same late-night hours as in previous years, albeit at higher levels. Black and Hispanic boys and men who were unemployed were significantly more likely to experience firearm injuries during the pandemic. Given that gun violence is an acknowledged public health epidemic that has long predated the COVID-19 pandemic, our results highlight the disproportionate consequences for vulnerable populations, such as Black and Hispanic communities as well as those with lower socioeconomic status, and the ongoing need for policies and procedures aimed at mitigating violence as a part of all-hazard preparedness.

## Notes

Supplement. **eTable 1.** Patients With Violent Penetrating Injury Presenting to Boston Hospitals, 2020 **eAppendix 1.** Analysis Measures **eAppendix 2.** Data Analysis Methods for ARIMA Model **eAppendix 3.** Data Analysis Methods for Trends During Surge Months April to October **eTable 2.** Annual Frequencies of Individuals With Penetrating Injuries Treated at Boston Medical Center, 2015-2021 **eFigure 1.** Kernel-Weighted Local Polynomial Regression of the 7-Day Mean of Gunshot Injuries Per Day Treated at Boston Medical Center **eFigure 2.** Trends in Day of the Week for Firearm Injuries Treated at Boston Medical Center Between April and October, 2015-2020 **eTable 3.** Patient and Injury Characteristics of Firearm Injuries Treated at Boston Medical Center Between April and October, 2015-2019
