# Supplementary material for: Trends in Violent Penetrating Injuries During the First Year of the COVID-19 Pandemic
Source: JAMA Netw Open. 2022 Feb 8;5(2):e2145708. doi: 10.1001/jamanetworkopen.2021.45708 (PMC8826178; doi:10.1001/jamanetworkopen.2021.45708)
Supplement: Supplement. — eTable 1. Patients With Violent Penetrating Injury Presenting to Boston Hospitals, 2020 eAppendix 1. Analysis Measures eAppendix 2. Data Analysis Methods for ARIMA Model eAppendix 3. Data Analysis Methods for Trends During Surge Months April to October eTable 2. Annual Frequencies of Individuals With Penetrating Injuries Treated at Boston Medical Center, 2015-2021 eFigure 1. Kernel-Weighted Local Polynomial Regression of the 7-Day Mean of Gunshot Injuries Per Day Treated at Boston Medical Center eFigure 2. Trends in Day of the Week for Firearm Injuries Treated at Boston Medical Center Between April and October, 2015-2020 eTable 3. Patient and Injury Characteristics of Firearm Injuries Treated at Boston Medical Center Between April and October, 2015-2019 [file jamanetwopen-e2145708-s001.pdf]

Supplemental Online Content

Pino EC, Gebo E, Dugan E, Jay J. Trends in violent penetrating injuries during the first year of the COVID-19 pandemic. *JAMA Netw Open*. 2022;5(2):e2145708. doi:10.1001/jamanetworkopen.2021.45708

eTable 1. Patients With Violent Penetrating Injury Presenting to Boston Hospitals, 2020

eAppendix 1. Analysis Measures

eAppendix 2. Data Analysis Methods for ARIMA Model

eAppendix 3. Data Analysis Methods for Trends During Surge Months April to October

eTable 2. Annual Frequencies of Individuals With Penetrating Injuries Treated at Boston Medical Center, 2015-2021

eFigure 1. Kernel-Weighted Local Polynomial Regression of the 7-Day Mean of Gunshot Injuries Per Day Treated at Boston Medical Center

eFigure 2. Trends in Day of the Week for Firearm Injuries Treated at Boston Medical Center Between April and October, 2015-2020

eTable 3. Patient and Injury Characteristics of Firearm Injuries Treated at Boston Medical Center Between April and October, 2015-2019

This supplemental material has been provided by the authors to give readers additional information about their work.

© 2022 Pino EC et al. *JAMA Network Open.*

eTable 1. Patients With Violent Penetrating Injury Presenting to Boston Hospitals, 2020

	January 1 - March 12			March 13 – May 31			June 1 – August 31			September 1 – December 31			2020
**Hospital**	**GSW**	**SW**	**Subtotal (%)**	**GSW**	**SW**	**Subtotal (%)**	**GSW**	**SW**	**Subtotal (%)**	**GSW**	**SW**	**Subtotal (%)**	**Total (%)**
**Boston Medical Center**	**18**	**28**	**46 (51)**	**59**	**65**	**124 (58)**	**90**	**64**	**154 (61)**	**62**	**79**	**141 (54)**	**465 (57)**
Mass General Hospital	7	9	16 (18)	14	40	54 (25)	23	21	44 (17)	28	34	62 (24)	176 (21)
Brigham and Women’s	7	1	8 (9)	12	4	16 (7)	19	12	31 (12)	20	8	28 (11)	83 (10)
Tufts Medical Center	3	9	12 (13)	2	11	13 (6)	4	8	12 (5)	8	12	20 (8)	57 (7)
Beth Israel Lahey Health	5	3	8 (9)	3	4	8 (4)	9	4	13 (5)	4	8	12 (5)	40 (5)
Totals	40	50	90	90	124	214	145	109	254	122	141	263	821 (100)

GSW = gunshot wound; SW = stab wound.

Data Sources: Violence Intervention and Injury Prevention Programs at Boston Children’s Hospital, Boston Medical Center, Massachusetts General Hospital, Tufts Medical Center, Brigham and Women’s Hospital, and Beth Israel Deaconess Medical Center as of 12/31/20.

Boston Children’s Hospital treated 7 children/youth with gunshot wounds and stabbings between March 13 and December 31, 2020. Data not available from Carney Hospital.

© 2022 Pino EC et al. *JAMA Network Open.*

eAppendix 1. Analysis Measures

Age was analyzed as both a continuous variable and dichotomized by the median gunshot wound patient age of 27 years. Race/ethnicity was classified by self-report into five categories: White, Black, Hispanic, other (Asian and all other races) and those missing race/ethnicity information (as a separate category). Health insurance payer was classified into four categories: Medicaid/Medicare, private, no health insurance, and unknown insurance status. Housing status was categorized into: permanent home, homeless/group home, and unknown housing status, based on patients' self-report. Homeless housing status includes clients living on the street, in a shelter, at friends' houses, or unknown homeless location. Employment status was classified by self-report as either employed (including both reported and unreported employment) or unemployed (including those seeking or not seeking work, students, retirees, and those unable to work due to disability or immigration status). Variables with missing information (unknown) were included in the analysis. These variables can be assumed to be missing at random based on covariate-dependent missingness (CDM)[24] analysis given the independent variable of injury year (p=0.06). Injuries were categorized by injury type (gunshot wound, stab wound) and hospital disposition. Of those patients who were admitted to the hospital, we assessed the length of stay and discharge placement. Patients designated as having been re-injured are those known to have previously been treated for a violent penetrating injury at BMC starting from June 2006.

eAppendix 2. Data Analysis Methods for ARIMA Model

These models allowed for examination of changes in presenting firearm injuries while accounting for seasonal trends and autocorrelation of data points.[25,26] Observation months from March 2020 and onward were defined as the post-intervention period (n = 12); previous months were defined as the pre-intervention period (n = 60). First order differencing and seasonal differencing of the monthly data induced stationarity, which was confirmed through Dickey-Fuller tests.[27] Plots of the autocorrelation function and partial autocorrelation function were used to guide the selection of autoregressive and moving average terms into the ARIMA model. Several models were tested using information criteria (AIC, BIC) and residual plots to determine the most parsimonious model with a good fit that adequately controlled for autocorrelation and seasonality [final model: arima(3,1,1) sarima(0,1,1,12)].[26] Transfer function models were used to quantify the complex impact of the intervention, with model fit statistics (AIC, BIC) used to select the most appropriate form of a pulse and decay function.[26,28] Analyses were performed using the *arima* and *tstf* (a wrapper of the tsa package in R) commands in Stata.[29]

eAppendix 3. Data Analysis Methods for Trends During Surge Months April to October

Construction of confidence intervals for local mean smoothed graphs of the 7-day average of penetrating injuries per day was performed by computing standard errors obtained by taking a square root of the estimate of the conditional variance of the local polynomial estimator at each day of the year.[31]

© 2022 Pino EC et al. *JAMA Network Open.*

We compared the frequency of firearm injuries by time of day and by day of the week in the pandemic year to the means and 95% confidence intervals of years 2015-2019. Due to the overwhelming majority of injuries occurring late at night, the data were re-coded so that each day of the week begins at noon on that day and ends on noon the following day, e.g. injuries coded as occurring on a Saturday have triage times from noon on Saturday to noon on Sunday. To evaluate possible epidemiological changes in firearm injury during the pandemic year surge months, we compared patient and injury characteristics of firearm injury patients to those of patients in the same months during the previous five years. To establish an equivalence of certain variables across the 5-year comparator time period of 2015-2019, we also assessed significant differences in demographics and injury characteristics across these years. Categorical variables were compared using χ^2 tests, or Fisher's exact test for variables with <5 expected observations in individual cells. Continuous variables in 2020 were compared to the previous five years using the Wilcoxon rank sum test after rejection of the assumption of normality using the Shapiro–Francia test.[32] Continuous variables from each year of March, 2015 – February, 2020 were compared using the Kruskal-Wallis equality-of-populations rank test. For categories that included unknown values, we report results before and after excluding these values. All analyses were conducted using STATA 16[33], and all statistical tests used 2-sided $p<.05$ as the threshold for significance.

© 2022 Pino EC et al. *JAMA Network Open.*

eTable 2. Annual Frequencies of Individuals With Penetrating Injuries Treated at Boston Medical Center, 2015-2021

Characteristic	No. (%)						*P* value
	2015	2016	2017	2018	2019	2020	
**Patients, No.**	**411**	**397**	**401**	**360**	**373**	**441**	.06
**GSW**	181 (44.0)	177 (44.6)	178 (44.4)	155 (43.1)	146 (39.1)	221 (50.1)	.002
**SW**	230 (56.0)	220 (55.4)	223 (55.6)	205 (56.9)	227 (60.9)	220 (49.9)	.78

All values are frequencies and column percentages of injuries from March of the listed year through February of the following year. *p*-values are derived from the Wilcoxon rank sum non-parametric test comparing penetrating injuries per day of March 2020 – February 2021 with the previous five years. GSW = Gunshot wound, SW = Stab wound.

© 2022 Pino EC et al. *JAMA Network Open.*

eFigure 1. Kernel-Weighted Local Polynomial Regression of the 7-Day Mean of Gunshot Injuries Per Day Treated at Boston Medical Center

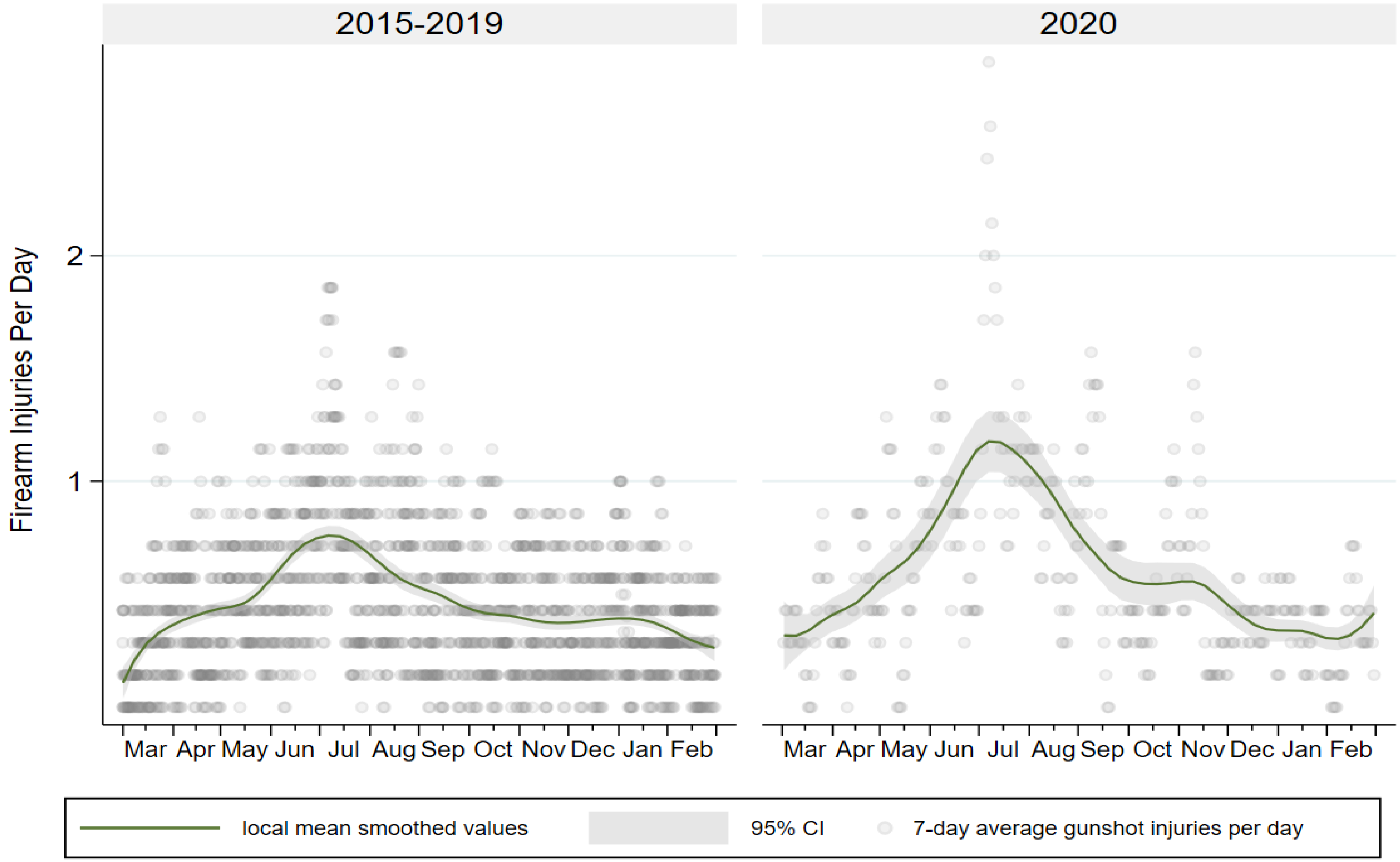

© 2022 Pino EC et al. *JAMA Network Open.*

eFigure 2. Trends in Day of the Week for Firearm Injuries Treated at Boston Medical Center Between April and October, 2015-2020

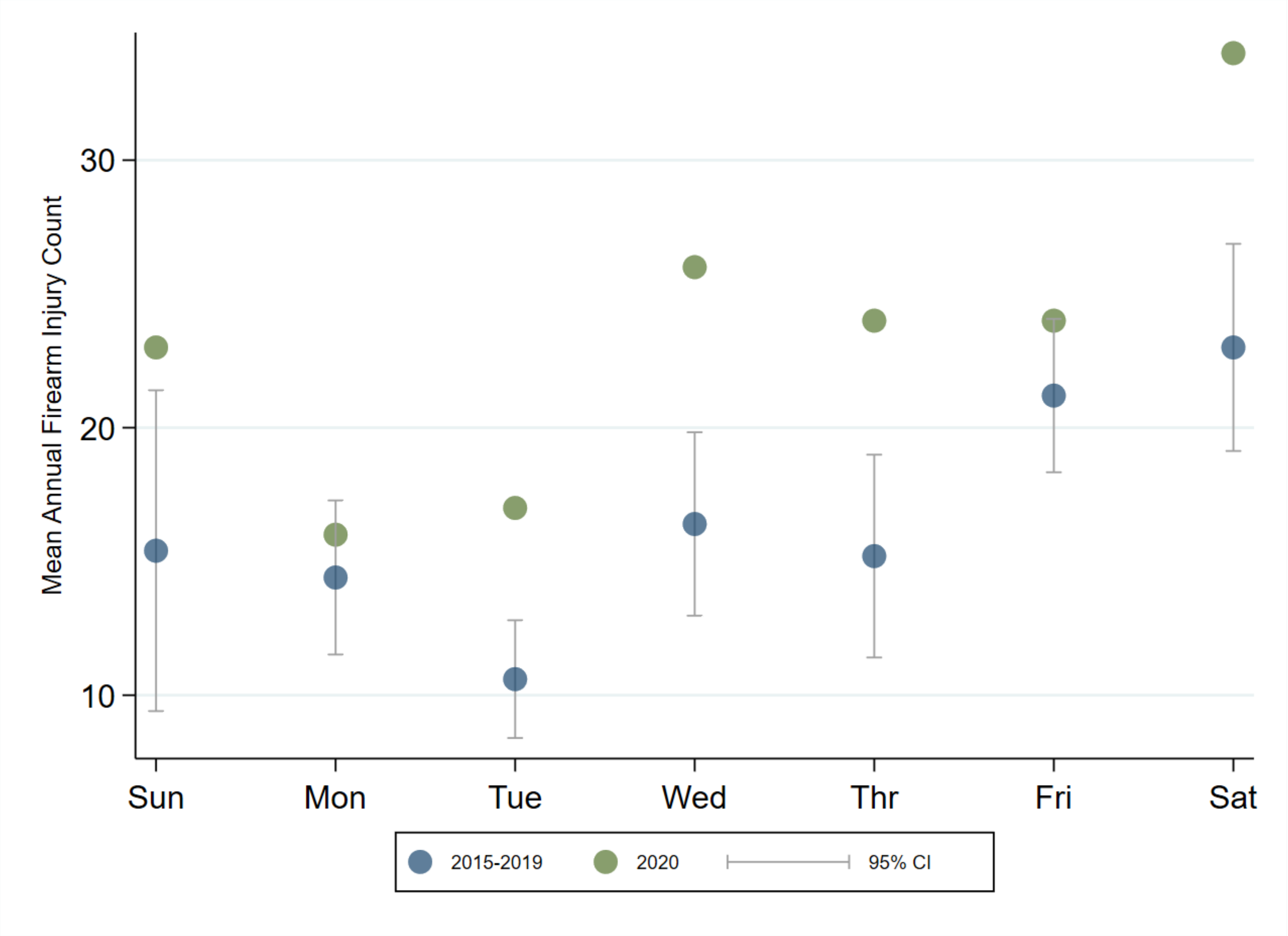

© 2022 Pino EC et al. *JAMA Network Open.*

eTable 3. Patient and Injury Characteristics of Firearm Injuries Treated at Boston Medical Center Between April and October, 2015-2019

**Year**	**2015**	**2016**	**2017**	**2018**	**2019**	***p***
**No. of injuries**	**127**	**112**	**130**	**104**	**108**	0.61
Age, median (IQR)	25.0 (9.7)	24.6 (10.9)	26.3 (12.5)	27.9 (11.1)	28.6 (11.9)	0.11
Age group						0.004
≤ 27	76 (59.8)	71 (63.4)	69 (53.1)	47 (45.2)	45 (41.7)	
≥ 28	51 (40.2)	41 (36.6)	61 (46.9)	57 (54.8)	63 (58.3)	
Gender						0.20
Male	112 (88.2)	93 (83.0)	111 (85.4)	97 (93.3)	96 (88.9)	
Female	15 (11.8)	19 (17.0)	19 (14.6)	7 (6.7)	12 (11.1)	
Race/ethnicity						0.02, 0.06[a]
Black	103 (82.4)	73 (68.9)	98 (77.8)	69 (70.4)	73 (75.3)	
Hispanic	18 (14.4)	24 (22.6)	21 (16.7)	23 (23.5)	13 (13.4)	
White	4 (3.2)	6 (5.7)	6 (4.8)	5 (5.1)	5 (5.2)	
Other[b]	0 (0.0)	3 (2.8)	1 (0.8)	1 (1.0)	6 (6.2)	
Unknown	*2*	*6*	*4*	*6*	*11*	
Insurance payer						0.002, 0.002[a]
Medicaid/ Medicare	68 (70.1)	67 (76.1)	81 (75.7)	40 (54.8)	44 (57.9)	
Private	11 (11.3)	14 (15.9)	12 (11.2)	15 (22.6)	22 (29.0)	
No health insurance	18 (18.6)	7 (8.0)	14 (13.1)	18 (24.7)	10 (13.2)	
Unknown	*30*	*24*	*23*	*31*	*32*	
Housing Status						<0.001, 0.04[a]
Permanent home	94 (89.5)	94 (93.1)	106 (90.6)	69 (79.3)	68 (87.2)	
Homeless / group home	11 (10.5)	7 (6.9)	11 (9.4)	18 (20.7)	10 (12.8)	
Unknown	22	11	13	17	30	
Employment Status						0.01, 0.12[a]
Employed	57 (56.4)	46 (47.9)	58 (50.0)	41 (49.4)	51 (65.4)	
Unemployed	44 (43.6)	50 (52.1)	58 (50.0)	42 (50.6)	27 (34.6)	
Unknown	26	16	*14*	*22*	*30*	
**Injury Specifics**						
Re-injury	29 (22.8)	18 (16.1)	24 (18.5)	22 (21.2)	18 (16.7)	0.64
Hospital Disposition						0.07
Admitted	92 (73.0)	91 (82.0)	99 (76.2)	72 (69.2)	77 (72.6)	
Discharged	28 (22.2)	16 (14.4)	25 (19.2)	17 (16.4)	18 (17.0)	
Eloped	1 (0.8)	3 (2.7)	2 (1.5)	4 (3.9)	2 (1.9)	
Deceased	5 (4.0)	1 (0.9)	4 (3.1)	11 (10.6)	9 (8.5)	
Missing	*1*	*1*	*0*	*0*	*2*	

© 2022 Pino EC et al. *JAMA Network Open.*

**Of those admitted:**											
Length of Stay, median (IQR)	1.9 (6.9)		1.7 (6.0)		3.4 (7.6)		4.0 (8.6)		3.6 (7.2)		0.06
Discharge Placement											0.71
Home	73 (81.1)		76 (83.5)		82 (82.8)		66 (91.7)		63 (81.8)		
Rehab/long term care/further hospitalization	14 (15.6)		7 (7.7)		11 (11.1)		4 (5.6)		7 (9.1)		
Left against medical advice	2 (2.2)		5 (5.5)		2 (2.0)		1 (1.4)		3 (3.9)		
Police custody	0 (0.0)		1 (1.1)		1 (1.0)		0 (0.0)		1 (1.3)		
Deceased	1 (1.1)		2 (2.2)		3 (3.0)		1 (1.4)		3 (3.9)		

All values are frequencies and column percentages except age and length of stay which are medians and interquartile ranges (IQR). Categorical variables were compared using χ^2 tests except for hospital disposition and discharge placement which were compared using Fisher's exact test. Continuous variables were compared using the Kruskal-Wallis equality-of-populations rank test. All *p*-values represent comparison of data from each year to all other years. Homeless housing status includes clients living on the street, in a shelter, at friends' houses, or unknown homeless location.

[a] *p*-value excluding unknown category

[b] 'Other' race includes Asian, American Indian or Alaska Native, Native Hawaiian or Other Pacific Islander

© 2022 Pino EC et al. *JAMA Network Open.*
